# Serum Hormone Levels in Female Patients With Atrophic Rhinitis

**DOI:** 10.7759/cureus.80309

**Published:** 2025-03-09

**Authors:** Sanghamitra Bhoi, Sujata Panda, Pranati Pradhan, Madhusmita Acharya, Sumitra Bhoi, Mamata Pandey, Satyabrata Meher, Binod K Sahu, Bimal K Panda

**Affiliations:** 1 Department of Biochemistry, Veer Surendra Sai Institute of Medical Science and Research, Sambalpur, IND; 2 Department of Ear, Nose, and Throat, Veer Surendra Sai Institute of Medical Science and Research, Sambalpur, IND; 3 Department of Obstetrics and Gynecology, Veer Surendra Sai Institute of Medical Science and Research, Sambalpur, IND; 4 Multi-disciplinary Research Unit, Veer Surendra Sai Institute of Medical Science and Research, Sambalpur, IND; 5 Department of Pathology, Veer Surendra Sai Institute of Medical Science and Research, Sambalpur, IND; 6 Department of Anesthesiology, Veer Surendra Sai Institute of Medical Science and Research, Sambalpur, IND

**Keywords:** atrophic rhinitis, estrogen, female, ozena, progesterone

## Abstract

Introduction: Atrophic rhinitis is a long-term inflammation of the nasal lining that leads to tissue thinning, abnormal cell changes, and crust buildup due to changes in blood vessel structure. It can be classified as primary or secondary, often developing after major nasal surgeries like inferior turbinectomy or as a result of infections such as tuberculosis, leprosy, or syphilis. The condition is marked by unusually wide nasal passages, nosebleeds, and, in rare cases, maggot infestation. The precise cause of primary atrophic rhinitis remains unclear. However, poor nutrition, low socioeconomic conditions, and inadequate hygiene have been linked to its development, particularly in association with *Klebsiella ozaenae*. It is more frequently observed in women of reproductive age. Research on hormonal influences in atrophic rhinitis has yielded mixed findings, and studies specifically examining hormone levels in this condition are relatively scarce.

Objective: This study aims to examine the association between female hormones, specifically estrogen and progesterone, nutritional status, and the development of atrophic rhinitis.

Materials and methods: This cross-sectional study was conducted on 60 female patients (aged 15-50 years) diagnosed with primary atrophic rhinitis in the Department of ENT and compared with 60 age-matched healthy female controls (15-50 years). Serum samples were collected, and estrogen and progesterone levels were estimated using ELISA (Merilyzer EIAQuant, Meril Life Sciences Pvt. Ltd., Vapi, Gujarat, India) at the Multi-disciplinary Research Unit, Veer Surendra Sai Institute of Medical Sciences and Research (VIMSAR), Burla. Following standard procedures, nutritional factors, including serum vitamin D3, vitamin B12, protein, iron, and calcium levels, were measured at RDC, VIMSAR, and Burla. Statistical analysis was performed using SPSS Statistics version 21.0 (IBM Corp. Released 2012. IBM SPSS Statistics for Windows, Version 21.0. Armonk, NY: IBM Corp.). A p-value <0.05 was considered statistically significant.

Results: This study found that serum estrogen levels (pg/ml) were significantly lower (p<0.0001) in cases compared to controls across all age groups. However, there was no statistically significant difference (p>0.05) in progesterone levels (ng/ml) between cases and controls across all age groups. Additionally, the mean serum levels of vitamin D3, vitamin B12, protein, iron, and calcium were significantly lower (p<0.05) in cases compared to controls.

Conclusion: This study suggests an association between low estrogen levels, poor nutritional status, and primary atrophic rhinitis in female patients.

## Introduction

Atrophic rhinitis is a chronic nasal disease characterized by a triad of symptoms: foul odor, nasal mucosal atrophy, and the formation of crusts due to periarterial fibrosis and endarteritis [1]. It may be primary or secondary to extensive nasal procedures such as inferior turbinectomy, polypectomy, and extensive rhinosporidiosis, or it may result from specific infections such as TB, leprosy, syphilis, and other granulomatous diseases. This disease is characterized by abnormally roomy nasal cavities, epistaxis, and occasional maggot formation, as described by Fraenkel [1]. It is a chronic, debilitating, and recalcitrant disease of the nasal cavities prevalent in several parts of the world [2].

Demographic and historical data reveal a female-to-male ratio of 5.6 to 1. However, there is no evidence of a hereditary component associated with atrophic rhinitis [3]. Estrogens have been considered a cause of nasal obstruction during the menstrual cycle and pregnancy [4]. Atrophic rhinitis is more prevalent among individuals with lower socioeconomic status and poor hygiene [5]. It has also been linked to developmental abnormalities, such as inadequate airflow in the maxillary sinuses, naturally large nasal cavities, or platyrrhine [2,6]. Several studies have reported nutritional deficiencies, particularly of vitamin A and iron, as possible contributing factors to atrophic rhinitis [7].

Ghosh conducted a study on one hundred cases of primary atrophic rhinitis. Radiological findings showed osteolysis in the turbinates even before significant clinical atrophy occurred. This was followed by the collapse of the turbinates due to osteolysis, leading to infection, crusting, and foul odor [8]. Additional infectious agents linked to atrophic rhinitis include *Coccobacillus foetidus ozaenae*, *Bacillus mucosus*, *Diphtheroids bacillus*, *Bacillus pertussis*, *Haemophilus influenzae*, *Pseudomonas aeruginosa*, and *Proteus* species [9]. Pathogen interaction occurs before mucus production and epithelial injury, and ciliostasis develops in the early stages within host tissue [10].

Several factors have been proposed as contributors to the development of atrophic rhinitis, including iron deficiency anemia, autoimmune diseases, chronic sinusitis, hormonal imbalances, low nutritional status, and certain infections [11]. The prevalence of primary atrophic rhinitis may have increased due to the excessive use of antibiotics for chronic nasal infections, while secondary atrophic rhinitis results from trauma, surgery, granulomatous diseases, infection, and radiation exposure [12]. Initially, bacterial contamination of the nasal environment led to olfactory impairment, prompting surgical intervention, which improved clinical parameters without directly affecting olfaction [13].

The diagnosis of atrophic rhinitis is primarily clinical, based on the symptom triad of foetor, greenish crusts, and a roomy nasal cavity. However, this full-blown clinical picture is usually observed in the later stages of the disease. In the early stages, only thick, viscid nasal discharge and loss of smell sensation may be present. Our study aims to explore any association between the occurrence of this disease and female hormonal status, as well as the nutritional status of patients attending our hospital.

## Materials and methods

This cross-sectional study was conducted in the Department of ENT, Department of Biochemistry, and the Multi-disciplinary Research Unit (MRU) at Veer Surendra Sai Institute of Medical Sciences and Research (VIMSAR), Burla, from September 2023 to December 2024. Sixty randomly diagnosed cases of primary atrophic rhinitis from the Department of ENT were recruited and compared with 60 age-matched healthy controls (15-50 years). Pre-designed, pre-tested questionnaires were used for sociodemographic data collection. The study was conducted after obtaining informed consent from the patients.

Ethical approval

The Institutional Ethical Committee of Veer Surendra Sai Institute of Medical Sciences and Research, Burla, approved the study protocol (approval number: 173-2022/I-F-O/32, approval date: 05.08.2022).

Inclusion and exclusion criteria

All clinically diagnosed female cases of primary atrophic rhinitis, aged 15-50 years, who attended the ENT OPD during the study period were included as cases. A healthy control group, which showed no signs of the illness, was selected to be representative of the population in terms of age, gender, and socioeconomic level. Male patients, those with secondary atrophic rhinitis, pregnant women, and prepubertal or postmenopausal females were excluded from the study.

Sample size

The sample size is calculated as follows:

$$n = \frac{(Z_{1-\alpha/2})^2 \cdot p \cdot q}{d^2}$$

where n is the desired sample size; Z1−α/2 is the critical value and a standard value for the corresponding level of confidence (at 95% confidence interval (CI) or 5% level of significance (type-I error), it was 1.96); P is the expected prevalence or based on previous research; q is 1 − p, and d is the margin of error or precision.

Assuming a prevalence of atrophic rhinitis was 1% [2], a precision of 3%, and a nonresponse rate of 30%, the sample size was estimated to be 57.2, which was rounded to 60.

Biochemical analysis

Two milliliters of venous blood were aseptically collected from ENT outpatient and inpatient patients. After centrifugation, blood serum was collected, and estrogen and progesterone levels were assessed using a fully automated ELISA (Merilyzer EIAQuant, Meril Life Sciences Pvt. Ltd., Vapi, Gujarat, India) following the manufacturer's guidelines at MRU, VIMSAR, Burla. Vitamins D3 and B12 were estimated using a CLIA analyzer (Electra FA, Tulip Diagnostics (P) Ltd., Goa, India) at RDC, VIMSAR, Burla. Serum protein and serum iron levels were measured using the Cobas Integra 400 plus analyzer (Roche Diagnostics International AG, Rotkreuz, Switzerland). In contrast, serum calcium was estimated using an Electrolyte Analyzer with an ion-selective chemiluminescence method (Ortho-Clinical Diagnostics, Inc., Rochester, New York, USA) at RDC, VIMSAR, Burla.

For BMI calculation, the weight and height of the subjects were measured, and BMI was determined using the Quetelet index as body weight (kg) divided by height (m²). BMI was used to define overweight and obesity [14].

Statistical analysis

Recorded data were collected in an Excel sheet (Microsoft Corporation, Redmond, WA, USA). Quantitative data were expressed as the mean ± SD, while qualitative data were expressed as frequency and percentage. A Student's t-test was performed to compare the case and control groups using SPSS Statistics version 21.0 (IBM Corp., 2012. IBM SPSS Statistics for Windows, Version 21.0. Armonk, NY: IBM Corp.). A p-value of <0.05 was considered statistically significant.

## Results

The socio-demographic characteristics according to the Kuppuswamy scale are shown in Table 1. Our study included 60 patients, with ages ranging from 15 years to over 45 years. The mean age was 29.9 ± 7.70 years in the control group and 30 ± 12.93 years in the case group. A higher percentage of respondents belonged to the lower socioeconomic status; in the case group, this was 51 (85%), while in the control group, it was 37 (61.66%). The highest proportion of participants with a normal BMI (kg/m²) was in the control group at 45 (75%), whereas underweight patients were more prevalent in the case group at 28 (46.67%). The majority of subjects in both groups belonged to the Hindu community, with 49 (81.67%) in the case group and 45 (75%) in the control group.

**Table 1:** Sociodemographic parameters of the study groups BMI: body max index, WHO: World Health Organization

Parameters	Control (N=60) %	Case (N=60) %
Age		
15-25	8 (13.33)	5 (8.33)
26-35	25 (41.67)	20 (33.33)
36-45	17 (28.33)	31 (51.67)
>45	10 (16.67)	4 (6.67)
Mean age in year	29.9 ± 7.70	30 ± 12.93
Socioeconomic status		
High	13 (21.67)	3 (5)
Middle	10 (16.67)	6 (10)
Low	37 (61.66)	51 (85)
BMI (kg/m^2^) (WHO Indian standard)		
Normal or less (18.5 ≤ 24.9)	45 (75)	27 (45)
Overweight (≥25-29.9)	12 (20)	2 (3.33)
Obese (≥30)	3 (5)	3 (5)
Underweight (≤18.5)	0 (0)	28 (46.67)
Religion		
Hindu	45 (75)	49 (81.67)
Muslim	8 (13.33)	6 (10)
Christian	6 (10)	4 (6.67)
Other (Sikh)	1 (1.67)	1 (1.66)

The most common symptoms among all 60 patients (100%) were the formation of crusts and nasal foetor. Other presenting complaints included loss of sense of smell in 52 patients (86.67%), nasal obstruction in 55 patients (91.67%), and nasal bleeding in 42 patients (70%), as shown in Table 2. Additionally, septal perforation was observed in two patients (3.33%), maggots were present in six patients (10%), and bilaterality was noted in 43 patients (71.67%), as also depicted in Table 2.

**Table 2:** Distribution of patients as per the symptoms and sign of female patients of atrophic rhinitis

Type of symptoms/sign	Case (N=60)	Percentage (%)
Nasal foetor	60	100
Crust formation	60	100
Loss of sense of smell	52	86.67
Nasal obstruction	55	91.67
Bleeding from nose	42	70
Headache	28	46.67
Maggots	06	10
Anosmia/hyposmia	33	55
Septal perforation	2	3.33
Bilaterality of diseases	43	71.67

In our study, the majority of patients, 42 patients (70%), had symptoms for a period of less than two years. Eleven patients (18.33%) experienced symptoms for two to six years, while seven patients (11.67%) had symptoms for more than six years, as shown in Table 3.

**Table 3:** Duration of diseases of female patients of atrophic rhinitis

Duration of disease	N (%)
<2 years	42 (70)
2-6 years	11 (18.33)
>6 years	7 (11.67)

The mean estrogen level (pg/ml) was highest in the 15-25-year age group for both the case (29.98 ± 0.73) and control (320 ± 40.13) groups, while the lowest levels were observed in the >45-year age group, with 15.09 ± 8.82 in the case group and 101.88 ± 70.21 in the control group, as shown in Table 4. However, our findings indicate that estrogen levels tended to be lower across all age groups in the case group. Similarly, the mean progesterone level (ng/ml) was highest in the 15-25-year age group for both case and control study participants, at 7.91 ± 0.51 and 7.66 ± 0.42, respectively. In contrast, for participants aged >45 years, progesterone levels were lower in both the case (2.45 ± 1.11) and control (2.43 ± 1.23) groups, as depicted in Table 4. No significant difference in progesterone levels between the case and control groups was observed in our study.

**Table 4:** Estrogen and progesterone levels of female patients of atrophic rhinitis * Significant at p<0.05

Age (year)	Estrogen level (pg/mL)		p-value	Progesterone level (ng/mL)		p-value
	Case (mean ±SD)	Control (mean ±SD)		Case (mean ± SD)	Control (mean ± SD)	
15-25	29.98 ± 0.73	321.68 ± 51.64	<0.0001*	7.91 ± 0.51	7.66 ± 0.42	0.429
26-35	27.45 ± 2.08	301.14 ± 2.69	<0.0001*	7.26 ± 0.52	7.37 ± 0.47	0.912
36-45	20.66 ± 0.53	270.94 ± 0.60	<0.0001*	5.74 ± 0.46	5.34 ± 0.52	0.524
>45	15.07 ± 9.005	104.86 ± 60.19	<0.0001*	2.45 ± 1.11	2.43 ± 1.23	0.915

The nutritional status, including vitamin D3 (ng/ml) 18 ± 3.65, vitamin B12 (pg/ml) 152.01 ± 55.44, calcium (mg/dl) 8.13 ± 0.90, protein (g/dl) 6.14 ± 0.40, and serum iron (µg/dl) 35.05 ± 6.13, was lower than the normal levels in the case group. A significant difference was observed between the case and control study groups in all nutritional parameters (p<0.0001), as shown in Table 5.

**Table 5:** Nutritional status of female patients of atrophic rhinitis * Significant at p<0.05

Parameters	Case (mean ± SD)	Control (mean ± SD)	p-value
Vitamin D_3_ (ng/ml)	18 ± 3.65	53.47 ± 9.31	<0.05*
Vitamin B_12 _(pg/ml)	152.01 ± 55.44	465.95 ± 58.20	<0.05*
Calcium (mg/dl)	8.13 ± 0.90	9.85 ± 0.58	<0.05*
Protein (g/dl)	6.14 ± 0.40	7.59 ± 0.34	<0.05*
Serum iron (µg/dl)	35.05 ± 6.13	112.61 ± 10.09	<0.05*

## Discussion

Atrophic rhinitis is a chronic nasal disorder characterized by degenerative changes in the nasal lining and the underlying bone structure [15]. Fraenkel was the first to describe the classical triad of symptoms, foetor, crusting, and atrophy of nasal structures, which is virtually diagnostic of the disease and is known as Fraenkel’s triad in his honor [16].

Our study included 60 patients with primary atrophic rhinitis. All cases were thoroughly investigated and showed no signs of leprosy, syphilis, TB, rhinoscleroma, or any other condition leading to secondary atrophic rhinitis. We included patients aged 15 to 50 years, representing the reproductive age group, with a mean age of 32.5 years. Most patients (31, 51.66%) belonged to the 36-45-year age group. In a study by Kanjikar et al. involving 190 cases, the prevalence of maggot-infested atrophic rhinitis was found to be 17.4%, with the highest occurrence in the 45-64 and >65-year age groups. The study also reported a notable female predominance, with a female-to-male ratio of 3.1:1. Rural residence and anemia were significant contributing factors to the increased prevalence [15].

Many researchers describe primary atrophic rhinitis as a disease of younger individuals. For instance, Sreedharan et al. found that 67% of cases (55 patients) were in the 11-30-year age group [17]. Similarly, in a study by Jain et al. examining the clinical characteristics of 62 atrophic rhinitis cases, the highest percentage (35.48%) occurred in the 11-20-year age group, with a male-to-female ratio of 1:1.06. The predominant symptoms included nasal crusting, nasal discharge, and an unpleasant nasal odor [18].

In our study, most patients (61.66%) had a low socioeconomic status. A similar observation was made by Bist et al. [19], who found that atrophic rhinitis was more prevalent among rural females due to poor hygiene, early marriage, low-calorie intake, and limited access to medical facilities. The patients' low socioeconomic status, unsanitary living conditions, and inadequate nutrition may have contributed to their susceptibility to disease development.

In our study, foul smells from the nose and crust formation were the most common symptoms in all patients (100%). The next most common complaints were loss of smell in 52 patients (86.66%), nasal obstruction in 55 patients (91.66%), epistaxis in 42 patients (70%), headache in 28 patients (46.66%), and maggots in six patients (10%). Somani et al. [20], in their study of 50 cases, also found that all patients (100%) had crust deposition, followed by ozaena in 70%, nasal blockage in 48%, epistaxis in 30%, anosmia in 28%, headache in 46%, nasal pain in 4%, and maggots in 20%.

Bist et al. [19] reported 90 primary atrophic rhinitis cases. Crusting of the nose was the most prevalent symptom, with mucosal atrophy, nasal crusting, and odor being the most consistent findings. Abnormalities in the sense of smell can manifest as anosmia or hyposmia. Anosmia may be present, and patients often become aware of the foul odor surrounding them only through the reluctance of others to come near them, an effect referred to as "merciful anosmia." Anosmia has been reported as a presenting feature in 40-90% of patients, as observed by Kedarnath and Mushtaq [21].

Our study found compromised smell sensation (anosmia/hyposmia) in 52 patients (86.66%). The degeneration of nerve endings leads to hyposmia or anosmia, depending on the severity of the disease. Septal perforation was observed in only two patients (3.33%). The disease was bilateral in 43 cases (71.66%). Kedarnath and Mushtaq [21] reported similar findings in their study of 20 cases, where 19 patients (95%) experienced loss of the sense of smell, two (25%) had septal perforation, and the disease was bilateral in 19 (10%) of cases. According to a study by Pampori et al., 80% of cases were bilateral [22].

Table 3 shows that most patients had a symptom duration of less than two years (70%), 18.33% of cases had symptoms lasting between two and six years, and 11.66% had symptoms for more than six years. However, it is well known that patients often become aware of their symptoms only after the disease has progressed significantly. Furthermore, they seek medical attention late due to lack health education and awareness. Sharan [23] found that good results were obtained in patients who started early treatment with shorter symptom duration.

Table 4 indicates that estrogen levels were low across all age groups, whereas progesterone levels remained similar to those in the control group. When comparing mean values, estrogen levels were significantly lower in all age groups (p<0.05), while progesterone levels were not statistically significant (p<0.05). Estrogens play proinflammatory and anti-inflammatory roles, depending on several factors, such as the type of immune response and the variability in the expression of different estrogen receptor isoforms [24]. High levels of estrogen promote anti-inflammatory effects by activating specific anti-inflammatory pathways. Elevated estrogen levels can also have an immunosuppressive effect, while low estrogen levels may exhibit immunostimulatory activity [25]. This suggests that hormonal stability plays a crucial role in immune response. In women, the menstrual cycle is primarily governed by ovarian hormones, estrogen, and progesterone [26], and their fluctuating levels depend on the biological responses of premenopausal women.

Table 5 shows that the mean serum levels of vitamin D3, vitamin B12, calcium, and serum iron were low in affected cases, while serum protein levels were within the normal range but on the lower side. This finding may be attributed to most patients from a low socioeconomic background with poor nutrition. Additionally, many patients had low BMI (28, 46.67%) and were underweight, compromising their immune response against infectious agents such as *Klebsiella ozaenae* and *Pseudomonas aeruginosa*. A study from Poland reported that ozaena commonly occurs in developing and underdeveloped countries where daily diets are deficient in iron, protein, and vitamins [11]. However, a study from Norway contradicted this finding by reporting a high prevalence of iron deficiency anemia without a correspondingly high incidence of atrophic rhinitis [27].

Strengths and limitations of this study

The assessment of our study was convenient due to its design. The study was conducted in a single tertiary center. Respondents' interpretations of the questionnaires varied from individual to individual. It is a cross-sectional study with a limited timeframe. Future large-scale studies with longer durations should be undertaken to validate the present study's findings.

## Conclusions

Our study found that most cases had lower estrogen levels than the control group, while progesterone levels showed no significant difference. Additionally, nearly all cases exhibited nutritional deficiencies, with the majority presenting a low BMI. These findings align with previous evidence suggesting that hormonal fluctuations may contribute to atrophic rhinitis, though standardized screening guidelines remain lacking. Recognizing these hormonal and nutritional associations and addressing deficiencies when necessary could help improve patient outcomes. Future research should explore the role of hormonal assessment in the diagnostic criteria and treatment protocols for atrophic rhinitis, potentially integrating endocrine and nutritional management strategies to enhance patient care and quality of life.
